# High Trapped Fields in C-doped MgB_2_ Bulk Superconductors Fabricated by Infiltration and Growth Process

**DOI:** 10.1038/s41598-018-31416-3

**Published:** 2018-09-06

**Authors:** A. G. Bhagurkar, A. Yamamoto, L. Wang, M. Xia, A. R. Dennis, J. H. Durrell, T. A. Aljohani, N. H. Babu, D. A. Cardwell

**Affiliations:** 1 Brunel Centre for Advanced Solidification Technology, Brunel University London, Uxbridge, UB8 3PH UK; 2 Department of Applied Physics, Tokyo University of Agriculture and Technology, 2-24-16 Nakacho, Koganei, Tokyo 184-8588 Japan; 3 Shanghai Jiao Tong University, 800 Dong Chuan Road, Shanghai, 200240 China; 4 Department of Engineering, University of Cambridge, Trumpington Street, CB2 1PZ Cambridge, UK; 5 National Centre for Advanced Materials, King Abdulaziz City for Science and Technology, Riyadh, 11442 Saudi Arabia

## Abstract

The grain boundaries in superconducting MgB_2_ are known to form effective magnetic flux pinning sites and, consequently, bulk MgB_2_ containing a fine-grain microstructure fabricated from nanoscale Mg and B precursor powders exhibits good magnetic field-trapping performance below 20 K. We report here that the trapped field of MgB_2_ bulk superconductors fabricated by an infiltration and growth process to yield a dense, pore-free microstructure, can be enhanced significantly by carbon-doping, which increases intra-band scattering within the superconducting grains. A maximum trapped field of 4.15 T has been measured at 7.5 K at the centre of a five-sample stack of Mg(B_1−*xi*_C_*xi*_)_2_ bulk superconductors processed by infiltration and growth, which not only represents a ~40% increase in trapped field observed compared to undoped bulk MgB_2_, but also is the highest trapped field reported to date in MgB_2_ samples processed under ambient pressure. The trapped field is observed to decay at a rate of <2%/day at 10 K, which suggests that bulk MgB_2_ superconductors fabricated using the infiltration and growth technique can be used potentially to generate stable, high magnetic fields for a variety of engineering applications.

## Introduction

A bulk superconductor can act effectively as a quasi-permanent magnet when magnetized by an applied magnetic field below its superconducting transition temperature, *T*_*c*_. Concentric supercurrents, induced typically throughout the bulk sample during the magnetisation process by the applied magnetic field, persist even when the external field is reduced to zero. The resulting, so-called trapped, magnetic field associated with these currents (the bulk material acts effectively as a thick, single-turn solenoid) decays extremely slowly, giving rise to a relatively stable magnetic field. Significantly, the magnitude of this trapped field is potentially much greater than that obtained typically using conventional Nd-Fe-B-based permanent magnets, which are limited generally to less than 2 T. Such compact superconducting bulk magnets are therefore excellent candidates for engineering applications where a high magnetic field is desired, such as desktop nuclear magnetic resonance (NMR), magnetic resonance imaging (MRI), motors and particle accelerators^1–3^.

MgB_2_ has been demonstrated to be a promising candidate for stable, high magnetic field applications. The key attributes from the perspective of practical applications are a combination of its high *T*_*c*_, which exceeds the predictions of BCS theory due to its peculiar two band nature^4^, and its tendency to form strongly linked grain boundaries, which allows the fabrication of polycrystals without the loss of inter-grain supercurrent^5,6^. As a result, a variety of methods have been developed and optimised to obtain dense, high performance MgB_2_ bulk superconductors, such as high pressure sintering^7–10^, spark plasma sintering^11^ and techniques based on infiltration^12,13^. In particular, infiltration and growth (IG), impregnation or reactive liquid infiltration (RLI) processes have been developed specifically to address challenges associated with conventional sintering routes, such as porosity and poor sinterability. In this approach, B powder is packed initially to form a green body, followed by infiltration with liquid Mg. The vapour infiltration route, used initially for processing MgB_2_ fibres of diameter 160 μm, was initially reported by Canfield *et al*.^14^ and later by Dunand *et al*. for the synthesis of Mg-MgB_2_ composites by infiltrating liquid Mg under pressure at 800 °C into a B preform^15^. The key advantages of this technique were demonstrated subsequently by Giunchi *et al*. (RLI) for the fabrication of state-of-the art bulk MgB_2_ artefacts^12,13^. Such a relatively simple, ambient pressure process not only results in the formation of hard, dense structures but can also be applied to fabricate complex geometries that are not achieved easily using conventional sintering techniques. Bulk MgB_2_ of relatively high density obtained by IG and RLI techniques is characterised typically by a high effective current carrying cross-sectional area, which translates directly to a higher critical current density (*J*_*c*_). Although *J*_*c*_ in self-field as high as 10^6^ A/cm^2^ has been measured in bulk samples, the performance of MgB_2_ tends to drop-off rapidly with applied magnetic field^16^. It is, therefore, essential that the in-field performance of bulk MgB_2_ is enhanced if Nb based low temperature superconducting materials used widely in existing practical applications and which typically require expensive liquid helium (LHe) as a coolant during operation, are to be replaced.

It is well understood that the performance of MgB_2_ can be enhanced by maximising the density of grain boundaries, which form effective pinning sites in the microstructure^17–21^. This has been achieved by employing nano-scale Mg and B precursor powders for the synthesis of MgB_2_, which results ultimately in the formation of fine-grained MgB_2_. Irradiation via the bombardment of neutrons, *γ* rays or heavy ions (Ag, Au) at sufficiently high velocities, which induces both large scale and small scale defects in the MgB_2_ lattice, is another effective approach for improving current carrying performance of bulk MgB_2_^22–24^. Large scale lattice defects, such as dislocations or stacking faults, act as effective pinning centres over a range of applied magnetic field and thereby improve the irreversibility field (*H*_*irr*_) of the superconductor (i.e. an extension of its ability to generate magnetic fields for practical applications at a given temperature). Small scale lattice defects, such as point defects, on the other hand, tend to reduce the mean free path of the superconducting charge carriers, which, in turn, reduces the coherence length and results in an increased upper critical field (*H*_*c2*_)^25^. Alternatively, a superconductor can be made “dirtier”, for example, by alloying to increase charge carrier scattering. Carbon doping (C-doping), in particular, has been shown to be a promising technique for enhancing *J*_*c*_*/H*_*c2*_ in bulk MgB_2_. Increased intra-band scattering (particularly in the *σ* band^26^), degradation of crystallinity^27–29^, enhanced vortex and *△k* pinning^30^, reduced anisotropy in critical fields^31^, strengthened grain boundary pinning^32^ are some of the mechanisms that have led to much improved *J*_*c*_*(B*) performance in C-doped MgB_2_ bulk superconductors. In addition, formation of defects in the microstructure such as stacking faults and nano-inclusions further contribute to pinning^33,34^. Since C in graphite allotropic form is difficult to dope into bulk MgB_2_^35^, it is often introduced in other forms that include SiC^33^, C-nanotube^36^, nano-diamond^37^ and organic compounds^38^.

In the present work, high quality Mg(B_1−*xi*_C_*xi*_)_2_ (where *x*_i_ is C occupancy on boron sites in MgB_2_ lattice) bulk superconductors were fabricated using an IG process, with B_4_C and SiC compounds as a source of C. Changes in the lattice parameters induced by carbon substitution were studied in these samples and their effects on the superconducting properties of the bulk material have been analysed in detail in an attempt to identify the level of C addition that yield optimum critical current density. The modified IG process (MPIG) was then adopted in order to facilitate uniform liquid Mg infiltration, which is necessary to produce homogeneous bulk MgB_2_ superconductors for practical applications. Finally, trapped field measurements were performed on the doped bulk samples and the results discussed.

## Experimental Methods

### Sample Preparation

Crystalline β-boron (98% pure, <40 μm) and B_4_C (99% pure, ~2 µm) powders were mixed using a mortar and pestle to obtain (100-*x*)% B - *x*% B_4_C (hereafter referred to as *x*% (B_4_C) precursor compositions with *x* varying in weight % as 0, 10, 20, 60 and 100 (Table 1). Sixth powder mixture containing β-boron and 10 wt% SiC (~30 nm) [referred to hereafter as 10% (SiC)], was also prepared, given that SiC addition had been demonstrated previously to yield effective C-doping in bulk MgB_2_^33^. Six precursor pellets of diameter 32 mm and thickness 6 mm, each weighing 7.5 g, were pressed uniaxially from these powder mixtures under a load of 35 MPa. The porous precursors were then subject to the IG process and reacted at 850 °C for 4 h, as described in refs^39,40^.

**Table 1:** Composition of the powders in the porous preforms used in IG and MPIG processes.

Nomenclature	IG		
	B (%)	SiC (%)	B_4_C (%)
Undoped	100	—	0
10% (B_4_C)	90	—	10
10% (SiC)	90	10	—
20% (B_4_C)	80	—	20
60% (B_4_C)	40	—	60
100% (B_4_C)	0	—	100
	**MPIG**		
	**B (%)**	**MgB**_**2**_ **(%)**	B_4_C (%)
Undoped MPIG	70	30	0
5% B_4_C (MPIG)	65	30	5
10% B_4_C (MPIG)	60	30	10

### Fabrication of High Performance, Homogeneous C-doped samples

The presence of continuous Mg channels in bulk MgB_2_ processed by IG has been known^40^. These non-superconducting channels, which form during the IG process, are typically several cm in size and obstruct the flow of supercurrent in the bulk microstructure, which, in turn, limits the field trapping ability of the sample. Therefore, in order to realize the advantages offered by C-doping on a larger scale, Mg channel free MgB_2_ bulk superconductors were fabricated by combining C-doping and a Modified Precursor Infiltration and Growth (MPIG) technique, as described in ref.^40^. The Addition of pre-reacted MgB_2_ phase powder to the boron precursor has been shown previously to facilitate uniform in-flux of Mg, which results in the formation of homogeneous, Mg-channel free microstructure in the bulk MgB_2_. As a result, appropriate mixtures of B, B_4_C and MgB_2_ powders were used as precursors in the MPIG process, as summarised in Table 1. 7.5 g of each precursor composition was pressed under a load of 35 MPa to obtain a pellet of diameter 32 mm and thickness 6 mm. Such C-doped bulk superconductors prepared using this method are referred to subsequently as *x*% B_4_C (MPIG), where *x* is the nominal weight % of B_4_C in the pellet prior to infiltration. Three bulk superconductors were fabricated by MPIG technique, one with *x* = 5, and two with *x* = 10, as described in Table 1.

### Characterization

XRD analysis (CuK_α_ = 1.5408 Å) was carried out in order to identify the phases present in the sample microstructure and to enable calculation of the crystallographic lattice parameters of the fully reacted compound. The non-uniform strain/distortion in the MgB_2_ lattice induced as a result of C substitution was quantified by Williamson-Hall (W-H) analysis^41^. The critical current density (*J*_*c*_) of these samples was calculated from the measured magnetic moment hysteresis loops using the extended Bean model for a rectangular cross-section (Sample dimensions ~ 3 × 3 × 1 mm^3^) in the presence of a magnetic field applied perpendicularly to the surface of the sample^42^.

### Trapped Field Measurements

The field-cooled (FC) method was used to magnetize the C-doped MgB_2_ bulk superconductors prior to the measurement of trapped magnetic field. This involved cooling down the sample, or sample arrangement, to 5 K in the presence of an external magnetic field of 5 T/6 T applied perpendicular to the top surface of the sample/samples using a Gifford–McMahon (GM) cryocooler (CRTHE05-CSFM, Iwatani Gas). The external magnetic field was then reduced to zero at a rate of 1.8 T/h to minimise any loss of trapped flux from the sample during the magnetization process (as shown in Supplementary Fig. 1). The sample was then heated slowly at a rate of 0.1 K/min and the trapped magnetic flux density measured at temperatures up to 40 K by cryogenic Hall sensors (HGCT-3020, Lake Shore).

Additional data related to this publication is available at the Brunel University London data repository (10.17633/rd.brunel.5395564).

## Results and Discussion

### Effect on *Tc*

**Figure 1:**
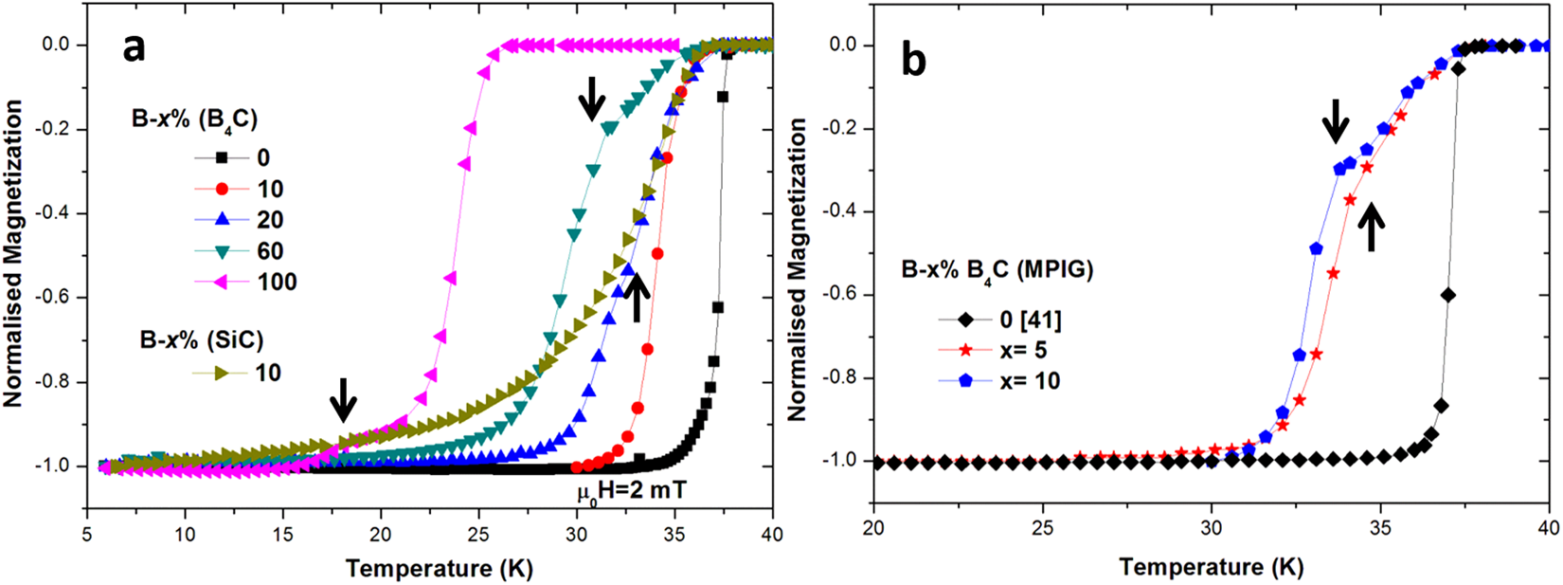
(**a**) Zero-field-cooled (ZFC) normalised magnetization as a function of temperature for IG samples *x*% (B_4_C) (*x:* 0, 10, 20, 60 and 100) and 10% (SiC). (**b**) ZFC normalised magnetization as a function of temperature for MPIG samples. The black arrows indicate 2^nd^ transition.

Figure 1(a) shows the normalised magnetization as a function of temperature for samples 10% (SiC), *x* = 10%, 20%, 60%, 100% (B_4_C) and an undoped sample. Both 10% (SiC) and 10% (B_4_C) resulted in a reduction of *T*_*c*_ (defined by onset of superconducting transition) from 37.9 K (undoped) to 36.2 K, arising from *σ* band filling by electron doping^43^. Electron doping reduces simultaneously the number of holes at the top of the *σ* bands and the electronic density of states^44^. Moreover, 10% (B_4_C) exhibited a relatively sharp superconducting transition, whereas 10% (SiC) showed a very broad transition, suggesting that C-doping is much less uniform in the sample containing SiC compared to that containing B_4_C. This can be explained by a homogeneous distribution of C at the atomic scale in the case of B_4_C. All the samples containing varying amounts of B_4_C exhibited a reasonably sharp superconducting transition with *T*_*c*_ reducing from 37.9 K (Undoped) to 25.9 K [100% (B_4_C)] as a result of C-doping in MgB_2_. Moreover, the addition of B_4_C with *x* ≥ 20 produced two distinct transition temperatures, suggesting possible presence of two types of Mg(B_1−*xi*_C_*xi*_)_2_ phases. The normalised magnetization as a function of temperature for MPIG samples is shown in Fig. 1(b). The MPIG samples, 5% B_4_C (MPIG) and 10% B_4_C (MPIG), exhibited a second transition at 34.8 K and 33.8 K respectively, below the onset *T*_*c*_ (37.8 K). Such high *T*_*c*_ onset of 37.8 K in MPIG samples suggests that C-doping has not taken place in the pre-reacted MgB_2_. The presence of the second transition at lower temperature (34.8 K and 33.8 K), however, suggests that higher levels of C doping into MgB_2_ that forms during the reaction of B with liquid Mg in the MPIG process.

### Phase analysis

**Figure 2:**
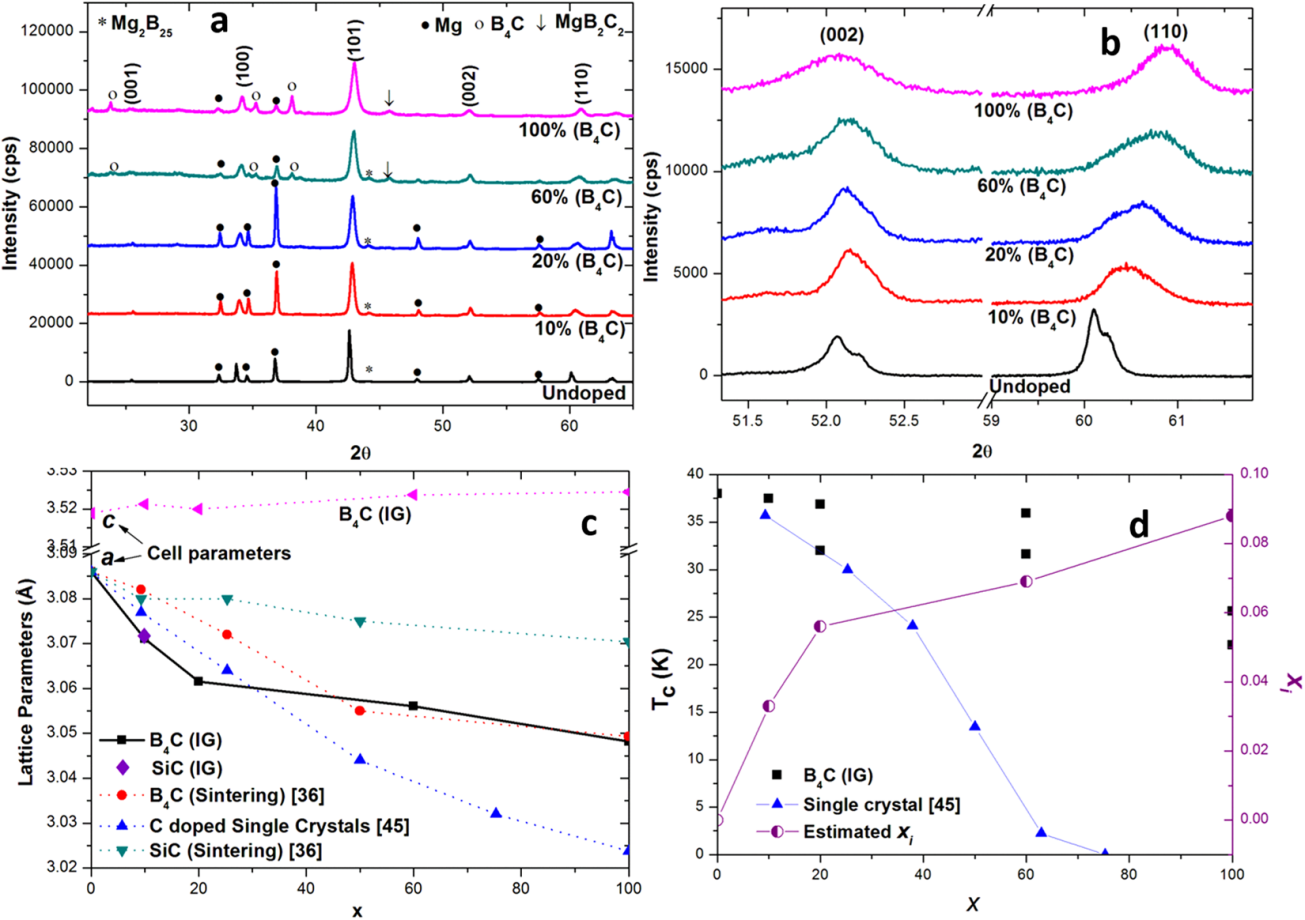
(**a**) XRD patterns for an undoped sample and B_4_C containing samples [*x*% (B_4_C)] revealing the presence of a majority Mg(B_1−*xi*_,C_*xi*_)_2_ phase along with minor concentrations of Mg, B_4_C and MgB_2_C_2_. (**b**) A significant shift observed in the (110) reflection while the (002) reflection remains almost unchanged. (**c**) Variation of the calculated lattice parameters *a/c* with C-doping. Data for sintered SiC/B_4_C containing and single crystal samples are also shown. (**d**) Measured values of *T*_*c*_ in C-doped samples carbon substitution (*x*_*i*_) as a function of nominal B_4_C content (*x*).

Figure 2(a) compares the XRD patterns obtained for B_4_C containing samples [*x*% (B_4_C)] with an undoped sample. The undoped sample shows the presence of familiar phases: MgB_2_, residual Mg and a small amount of metastable Mg_2_B_25._ On the other hand, the samples containing B_4_C are composed predominantly of C-doped, superconducting Mg(B_1−*xi*_,C_*xi*_)_2_. The equilibrium MgB_2_C_2_ phase and a small amount of unreacted B_4_C were detected for higher nominal B_4_C content of *x* ≥ 60%. Figure 2(b) provides insight into nature and extent of C-doping in MgB_2_. The peaks for the B_4_C containing samples exhibit a component of a plane normal to the crystallographic *a-b* plane, and show a consistent and significant shift towards higher angles with considerable peak broadening. In addition, the peak positions corresponding to planes parallel to the basal (001) plane remain almost unchanged. This clearly suggests, and is in good agreement with previously observed studies^33,35^, that C-doping induces strain in the crystallographic *a-b* plane only.

Figure 2(c) shows the calculated in-plane lattice parameter for MgB_2_ as a function of B_4_C addition [*x*% (B_4_C)]. The data obtained are compared with those for C-doped MgB_2_ single crystals and for C-doped samples produced by B_4_C and SiC via a sintering route under similar reaction conditions^44^. It is apparent from the change in lattice parameter ‘*a*’ that B_4_C is a more efficient dopant than SiC. Also, the C-doping level for IG samples with 10% (B_4_C) and 20% (B_4_C) appears to be higher than that observed in C-doped single crystals. This could be due to the fact that about 15% boron is present in the form of the intermediate boride phase, Mg_2_B_25_, which is not transformed fully into superconducting MgB_2_^39^. This possibly results in an apparent increase in carbon concentration in MgB_2_. Expectedly, A small change in lattice parameter ‘*c*’ was observed from 3.518 to 3.524 Å from the undoped sample to 100% (B_4_C) sample. Figure 2(d) compares *T*_*c*_ observed in C-doped samples with nominal B_4_C addition [*x*% (B_4_C)]. The actual carbon substitution produced by B_4_C addition, which is estimated by comparing the lattice parameter ‘*a*’ obtained for the present samples with that for the single crystals of Mg(B_1−*xi*_C_*xi*_)_2_ shown in Fig. 2(c), is also presented. The maximum carbon substitution on the B site of up to *x*_*i*_ = 0.088 was obtained for the samples containing B_4_C. The effective carbon substitution (*x*_*i*_) in 5% (B_4_C) MPIG and 10% (B_4_C) MPIG samples was estimated to be 0.022 and 0.033 respectively.

The reaction of B_4_C with Mg is known to result in the formation of MgB_2_ via the following chemical reactions:

I $$Mg(l)+{B}_{4}C+B\to Mg{({B}_{1-{x}_{i}}{C}_{{x}_{i}})}_{2}\,\,({x}_{i} < 0.15)$$

II $$Mg(l)+{B}_{4}C\to Mg{({B}_{0.85}{C}_{0.15})}_{2}+Mg{B}_{2}{C}_{2}\,\,\,({x}_{i} > 0.15)$$

Detailed analysis by Wilke *et al*.^45^ suggests that an equilibrium MgB_2_C_2_ phase begins to appear when carbon saturation is reached, i.e. when C can no longer be accommodated in the MgB_2_ lattice, which is precipitated subsequently in the form of MgB_2_C_2_. Analysis of C-doped MgB_2_ single crystals shows that C saturation occurs at about *x*_*i*_ = 0.15^44^. In the present study, the presence of MgB_2_C_2_ was detected for a B_4_C content of *x* ≥ 60 (*x*_*i*_ = 0.07), despite the fact that the actual substitution *x*_*i*_ was <0.15. This suggests that carbon saturation probably occurred locally within the microstructure. Moreover, the presence of residual B_4_C indicates that not all the carbon within the precursor composition doped into the MgB_2_ lattice.

**Figure 3:**
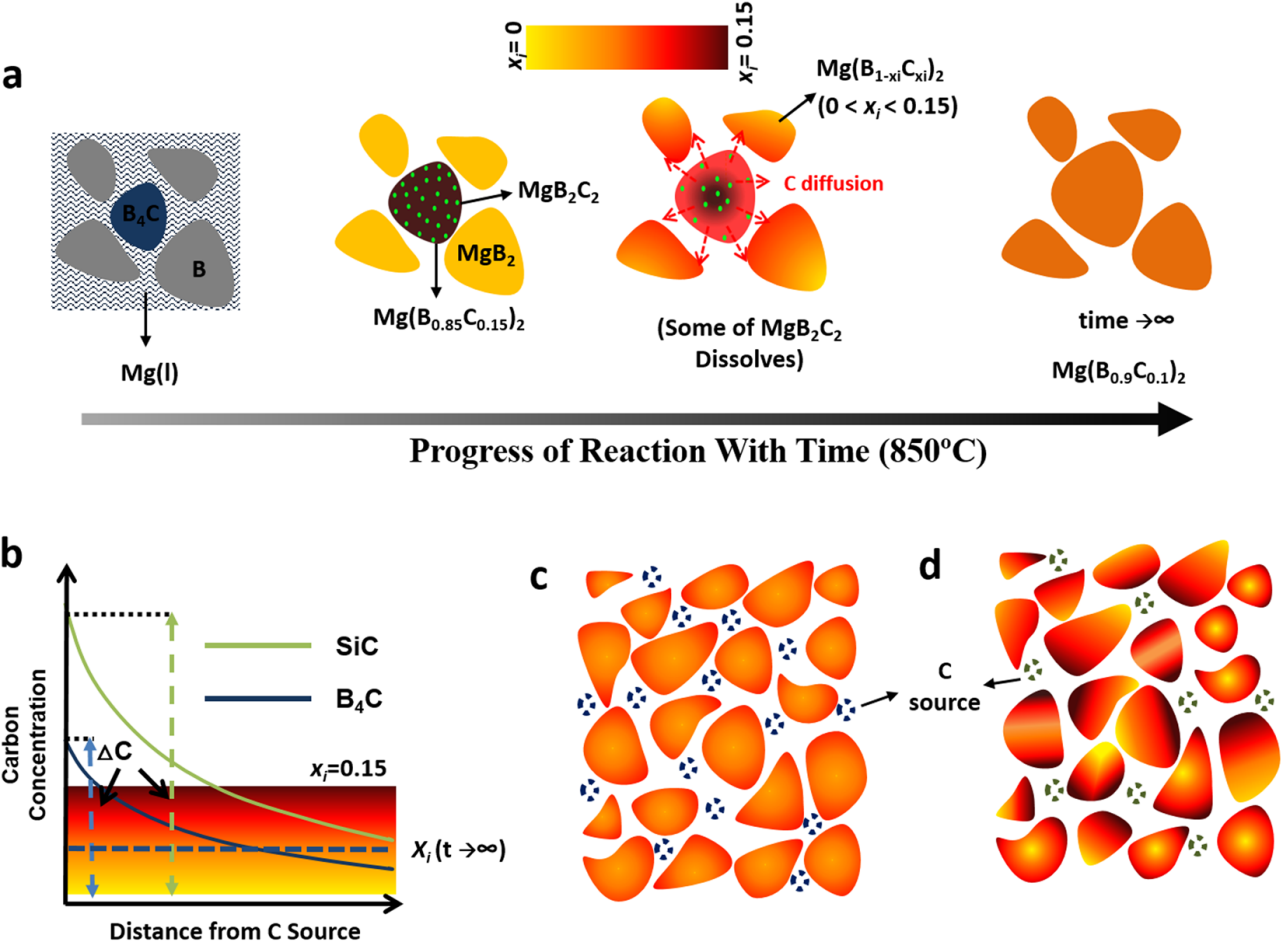
(**a**) Schematic illustration of C-doped MgB_2_ bulk superconductor processed by infiltrating liquid Mg into Boron precursor containing 10% B_4_C at 850 °C. The illustrations shown from left to right represent the various stages of reaction with time. The *x*_*i*_ value for 10% (B_4_C) is calculated to be 0.033. (**b**) Expected carbon concentration gradients originating from two different carbon sources. The relatively large difference in *ΔC* is due to a large variation in C at% between the two dissimilar SiC and B_4_C chemical phases. A higher degree of C variation within the Mg(B_*1-xi*_C_*xi*_)_2_ grain associated with (**c**) B_4_C addition relative to (**d**) SiC addition is observed. Carbon sources are represented by the dashed regions. Note that the intermediate boride formation during the transformation of B to Mg_2_B_25_ is not shown.

The mechanism of C-doping in MgB_2_ from B_4_C in the IG process can be understood from Figs 1 and 2, and is summarised in Fig. 3(a). Bulk Mg(l) infiltration occurs after 20–40 minutes from the beginning of the IG process^39^. This is followed by the start of the transformation of B and B_4_C particles into MgB_2_ and C-doped MgB_2_ (Reaction II), respectively. The excess carbon in B_4_C, which cannot be accommodated in the Mg(B_0.85_C_0.15_)_2_ lattice_,_ precipitates in the form of MgB_2_C_2_. The carbon then diffuses into neighbouring undoped MgB_2_ grains. Figure 3(b) shows the expected carbon concentration gradient from C-sources the B_4_C and SiC, respectively, assuming that both are added separately to B to achieve the same target *x*_*i*_ in the Mg(B_1-*xi*_C_*xi*_)_2_ phase when all the C is doped in Mg(B_1−*xi*_C_*xi*_)_2_ for an infinite reaction time. In this case, a steeper C-concentration gradient is expected since the atomic % of C is higher in SiC (0.5) than it is in B_4_C (0.2). This results conceivably in a wider spread in *ΔC* and a resulting broader *T*_*c*_ (Fig. 1). Furthermore, it is noted that the number of C-sources with SiC addition is significantly lower than those associated with B_4_C addition when the B_4_C and SiC particle sizes are comparable. This is because number of moles of SiC required to achieve a target *x*_*i*_ is fewer than the number of moles of B_4_C required to achieve the same level of doping, coupled with the smaller molar volume of SiC relative to B_4_C. This is likely to result in higher composition fluctuations of *x*_*i*_ in the SiC containing sample [Fig. 3(c)] compared to that containing B_4_C [Fig. 3(d)].

### Strain analysis

**Figure 4:**
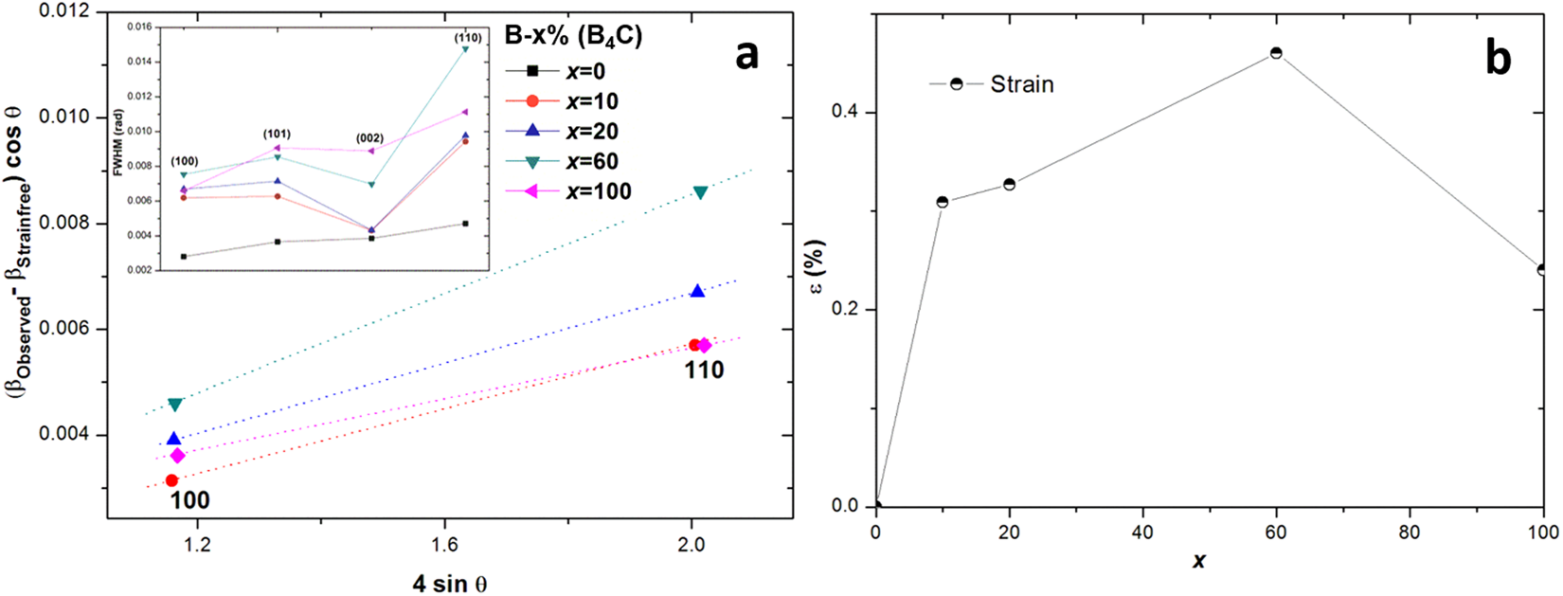
(**a**) A Williamson-Hall plot obtained from FWHM data for the major reflections (inset) in the XRD pattern for undoped and C-doped samples. (**b**) Non-uniform strain calculated from W-H plot as a function of nominal B_4_C content.

Figure 4(a) shows the full width at half maximum (FWHM) data for various XRD reflections as a function of nominal B_4_C content [*x*% (B_4_C)]. It is apparent that C-doping has induced a large amount of lattice strain/distortion and degradation of the crystallinity in MgB_2_, but mostly in the *a-b* direction, as evidenced by the increase in FWHM for the (100) and (110) reflections. In comparison, the FWHM of the (002) reflection appears rather small. The non-uniform strain/distortion in the MgB_2_ lattice can be quantified by a Williamson-Hall (W-H) plot according to following equation^41^:

1 $$({\beta }_{Observed}-\,{\beta }_{Instrumental})\cos \,\theta =\frac{K\,\lambda }{L}+4{\varepsilon }_{N}\,\sin \,\theta $$

where *β*_*Observed*_ and *β*_*Instrumental*_ are the observed and instrumental broadening in radian respectively, *K* is the Scherrer constant determined by crystallite size (~1), *λ* and *L* are the X-ray wavelength and size of diffracting domain in Å, respectively, and *ε*_*N*_ is the non-uniform strain. Here, *β*_*instrumental*_ is approximated to be the FWHM of an undoped sample, which is assumed to be strain-free. Also, since strain is present only in the *a-b* plane, only the (100) and (110) reflections are considered for the purposes of strain analysis. A W-H plot is shown for nominal B_4_C content in Fig. 4(a). The C-doping was found to induce strain of up to 0.46% in the lattice, as shown in Fig. 4(b). In addition, semi-coherent precipitates (MgB_2_-Mg) were detected in TEM analysis (Supplementary Fig. 2). Such a formation of semi-coherent interface further leads to localized strain in the interface region.

### *Jc* and Pinning Force

**Figure 5:**
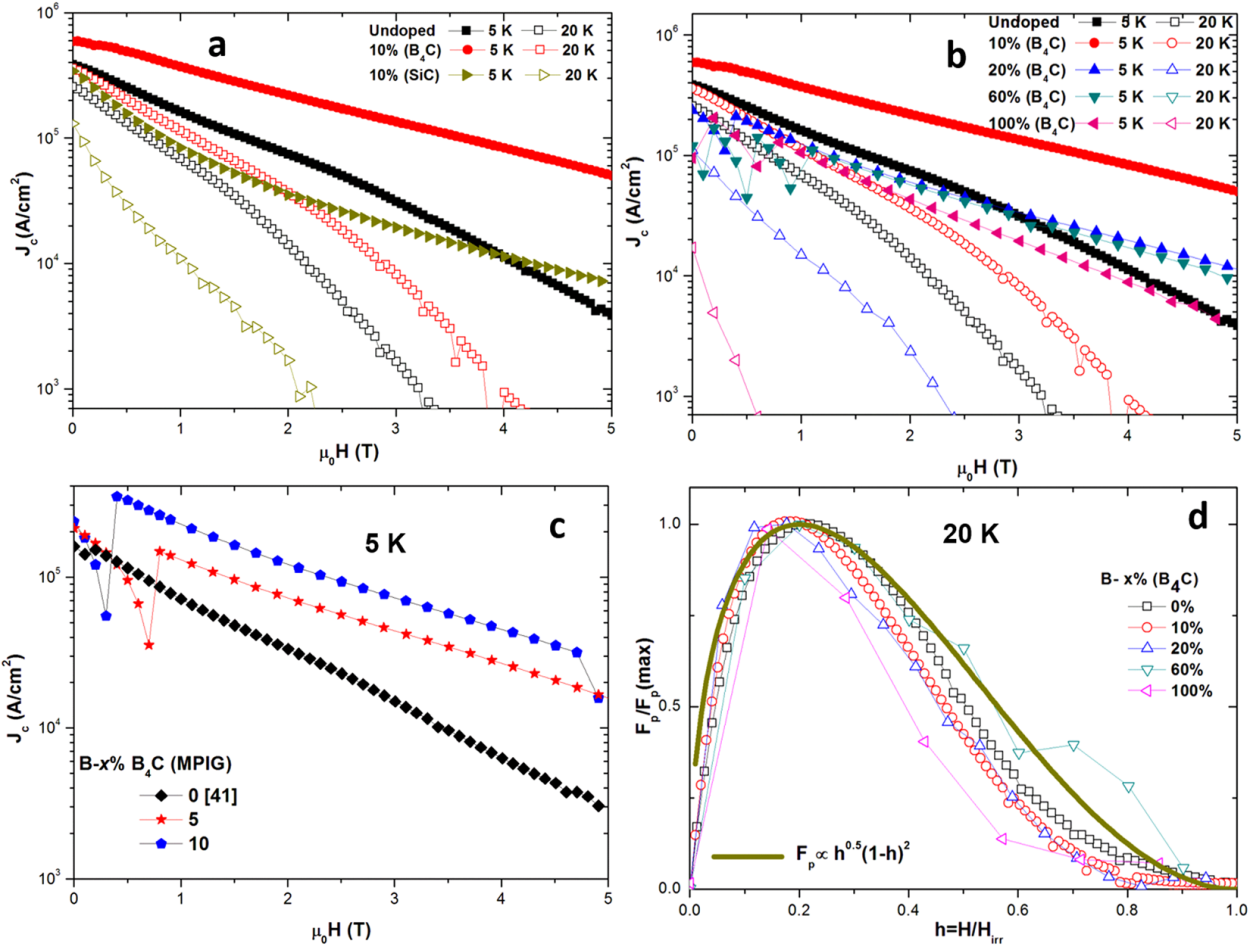
Measured *J*_*c*_ as a function of external field for samples (**a**) 10**%** (SiC), 10% (B_4_C) (**b**) and *x*% (B_4_C) from *x* = 0 to *x* = 100 and (**c**) *J*_*c*_ as a function of external fields at 5 K for MPIG samples. (**d**) Pinning force (*J*_*c*_*xB*) at 20 K, as a function of reduced field (*µ*_0_*H*/*µ*_0_*H*_*irr*_) for undoped and doped MgB_2_ samples processed by IG.

Figure 5(a) compares *J*_*c*_ at 5 and 20 K as a function of external field for 10% (SiC), 10% (B_4_C) and the undoped sample. It can be seen that the C-doped samples exhibit a much weaker dependence of *J*_*c*_ on external field, and particularly at 5 K. The sample containing B_4_C, 10% (B_4_C) in particular, shows a significantly enhanced self-field *J*_*c*_ of 600 kA/cm^2^ (5 K) and 350 kA/cm^2^ (20 K), which represents an increase of 50% and 36% with respect to the *J*_*c*_ measured in the undoped sample.

In addition, an in-field *J*_*c*_ (5 T, 5 K) as high as 50 kA/cm^2^ was observed for this sample, which is nearly 10 times that observed for the undoped sample. The enhanced flux pinning, (at higher external fields, in particular) and *H*_*irr*_ is clearly a result of induced strain in the lattice, or degradation in the crystallinity of MgB_2_. Such lattice imperfections enhance the intra-band scattering and reduce the electron mean free path *l* and coherence length *ξ* according to the following equation^46^:

2 $$\frac{1}{{\rm{\xi }}}=\frac{1}{{{\rm{\xi }}}_{0}}+\frac{1}{l}$$

A shortening of *ξ* allows more flux to be accommodated in the sample, which results in an increased *H*_*c2*_. On the other hand, the sample containing SiC did not exhibit a similar improvement in *J*_*c*_ due to intrinsic, inhomogeneous C-doping [Fig. 1(a)]. Moreover, the reaction of SiC with Mg is known to lead to the formation of non-superconducting Mg_2_Si inclusions in the bulk microstructure, which reduces the effective current carrying cross section of the sample^33,47^. Previous studies of C-doped MgB_2_ bulk samples have also reported improved *J*_*c*_*-B* behaviour. Nominal composition of MgB_1.8_(SiC)_0.1_ exhibited a two-fold increase in *J*_*c*_ (100 kA/cm^2^ at 3 T, 20 K) compared to undoped sample. Similarly, *J*_*c*_ in MgB_1.5_(B_4_C)_0.1_ exhibited a five-fold increase (50 kA/cm^2^ at 5 T, 5 K) compared to *J*_*c*_ in undoped sample^33^. Serquis *et al*. further reported high in-field *J*_*c*_ of up to 50 kA/cm^2^ (5 T, 5 K) for samples containing C-nanotube^36^ and a diamond doped sample showed an improved *J*_*c*_ of 10 kA/cm^2^ (20 K, 4 T), from an undoped reference *J*_*c*_ of 100 A/cm^2^ ^37^. A novel technique, which involved coating B with malic acid and toluene with B prior to sintering, resulted in an eight-fold increase in *J*_*c*_ to 40 kA/cm^2^ (20 K, 5 T)^38^.

In this study, *J*_*c*_*-B* was also measured for varying B_4_C content [*x*% (B_4_C)] in order to observe any change in *J*_*c*_ with C-doping, as shown in Fig. 5(b). 100% (B_4_C) exhibited a relatively high self-field *J*_*c*_ of 250 kA/cm^2^ at 5 K, despite having a low *T*_*c*_ (22 K). This, together with the superconducting transitions shown [Fig. 1(a)] and the XRD [Fig. 2(a)] suggests that B_4_C is not only highly reactive with Mg, but that the superconducting Mg(B_1-*xi*_C_*xi*_)_2_ phase is also well connected within the bulk microstructure. 10% (B_4_C) exhibited optimum *J*_*c*_*(B)* performance, with *J*_*c*_ degrading gradually for higher B_4_C content. This demonstrates a ‘trade-off’ between enhanced flux pinning and a reduction in *T*_*c*_ as a result of C-doping. Undoped MPIG, 5% (B_4_C) MPIG and 10% (B_4_C) MPIG samples, on the other hand, exhibited self-field *J*_*c*_*’s* of 200, 250 and 440 kA/cm^2^, respectively at 5 K as shown in Fig. 5(c). It is apparent that the MPIG samples show reduced *J*_*c*_ with respect to IG samples. For instance, 10% (B_4_C) and 10% B_4_C (MPIG) samples showed a maximum self-field *J*_*c*_ of 600 and 440 kA/cm^2^ respectively, at 5 K. This is because the grain boundary areas of such pre-reacted MgB_2_ particles are of low *J*_*c*_, due to weaker connectivity and lower *H*_*c2*_ in such areas, as suggested by the magneto-optical observations of Polyanskii *et al*.^48^.

The variation of normalised pinning force *F*_*p*_*/F*_*p*_*(max)* with reduced field (*h* = *H/H*_*irr*_) is shown in Fig. 5(d) for undoped and C-doped samples. *H*_*irr*_ is defined as the field at which *J*_*c*_ = 10^2^ A/cm^2^. Dew-Hughes proposed the following equation characterising pinning forces in type II superconductors that originate from various sources^49^:

3 $${F}_{p}(h)={F}_{p}/{F}_{p(max)}\propto {h}^{p}{(1-h)}^{q}$$

where the parameters *p* and *q* are material constants. Dew-Hughes suggested six different pinning mechanisms based on equation (3): (1) *p* = 0, *q* = 2: normal core pinning, volume pins; (2) *p* = 1, *q* = 1: *Δk*-pinning, volume pins (3) *p* = 1/2, *q* = 2: normal core pinning, surface pins; (4) *p* = 3/2, *q* = 1: *Δk*-pinning, surface pins; (5) *p* = 1, *q* = 2: normal core pinning, point pins; and (6) *p* = 2, *q* = 1: *Δk*-pinning, point pins.

The maximum of the normalized pinning force is expected at *h* = 0.2 for samples where surface pinning is dominant, such as that observed for undoped MgB_2_ in Fig. 5(d). Such pinning is also observed in Nb-based superconductors where grain boundaries form dominant pinning sites^50^. The apparent deviation of pinning force from Dew-Hughes model results probably from anisotropy in *H*_*c2*_, which is origin of *H*_*irr*_ in MgB_2_ rather than thermally activated depinning^51^. The normalised pinning force maxima in the samples containing B_4_C are shifted to lower fields at both 5 K and 20 K. For instance, 10% (B_4_C) and 20% (B_4_C) exhibit maxima at *h* = 0.18 and 0.15, respectively at 20 K. Such behaviour has also been reported by Matsushita *et al*.^52^, Cheng *et al*. in C-doped samples^36^ and Shcherbakova *et al*. in bulk MgB_2_ containing sugar^53^. This suggests the possible presence of other, non-surface, flux pinning centres in C-doped samples, such as volume pinning. Recently, Yang *et al*. also observed such shift for MgB_2_ containing Dy_2_O_3_^54^. Dy based nano-inclusions were considered to form additional pinning sites and the origin of the observed shift. In the present study, fine precipitates in the sample microstructure, such as MgB_2_C_2_ or unreacted B_4_C could contribute towards volume pinning. Such a contribution to pinning from inclusions was reported by Dou *et al*. in sintered Mg(B_1−*xi*_C_*xi*_)_2_ containing SiC^33^. Alternatively, such a shift in pinning force maxima towards lower field could also result from inhomogeneous C-doping, and a resultant distribution of *H*_*C2*_, within the bulk microstructure.

### High Performance homogeneous C-doped samples

Long range microstructural homogeneity and uniformity in the level and extent of carbon doping in the MgB_2_ bulk matrix plays a critical role in determining the flux trapping potential of this material, although *J*_*c*_ measured in a sample of dimensions of several mm gives a good indication of this at a more local level. As a result, homogeneous, defect free C-doped MgB_2_ bulk superconductors were fabricated by a MPIG process (Section 2.2). 5% B_4_C (MPIG) and 10% B_4_C (MPIG) samples were prepared in order to study the trapped field performance of bulk samples given that optimum *J*_*c*_*(B)* behaviour was obtained for 10% (B_4_C) [Fig. 5(b)].

#### Trapped Field Measurements

**Figure 6:**
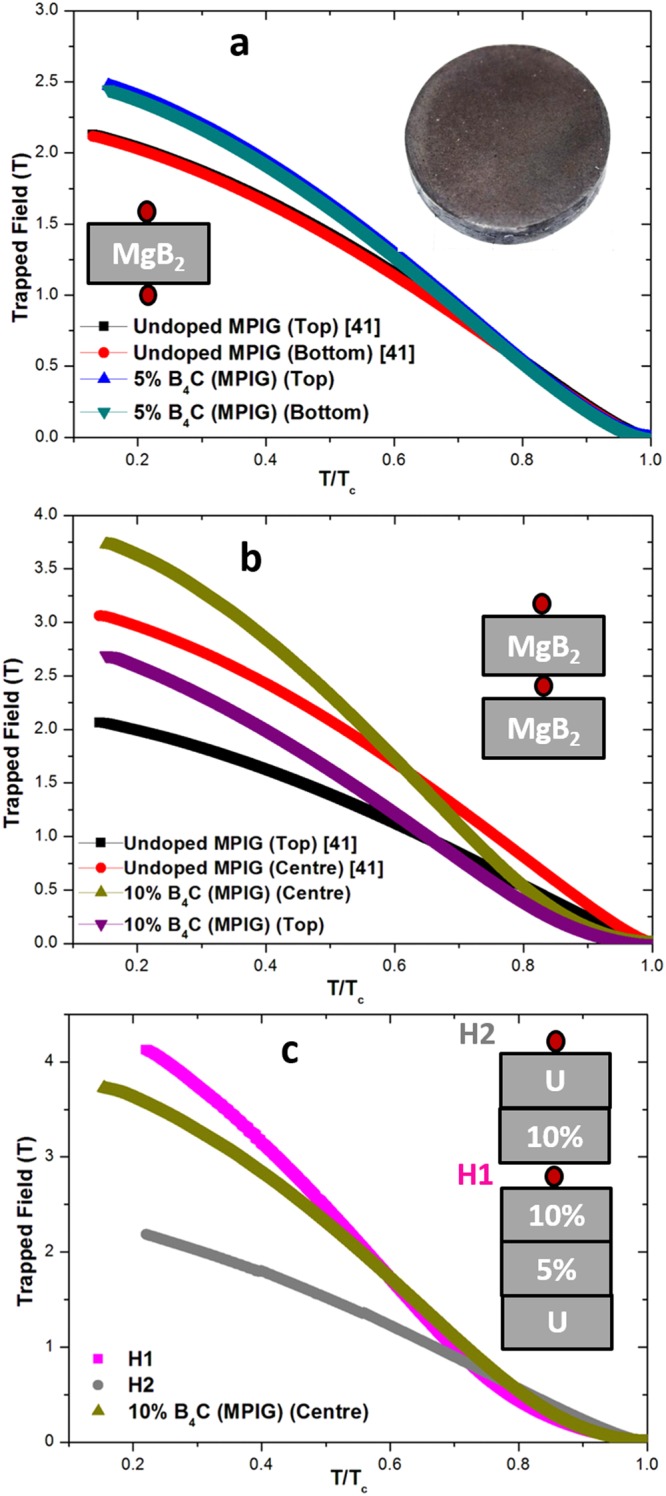
Maximum trapped field as a function of normalised temperature for (**a**) 5% B_4_C (MPIG) (**b**) 10% B_4_C (MPIG) bulk MgB_2_ superconducting discs. Data for undoped MPIG bulk samples measured in the same configuration are also shown (**c**) Trapped field dependence with normalised temperature for a stack arrangement (Stack-5). The location of the hall probe(s) is shown in the insets and an image of representative bulk shown in (**a**). H1 was placed in the middle of gap (1.6 mm) between the two bulks.

Three sets of measurements were performed as follows - (i) 5% B_4_C (MPIG) [Fig. 6(a)], (ii) a two-sample stack of 10% B_4_C (MPIG) [Fig. 6(b)] and (iii) a five-sample stack [Fig. 6(c)], referred to subsequently as Stack-5. The arrangement of bulk superconductors and the location of hall probes are shown in the respective insets in Fig. 6. For the purpose of comparison, the trapped field at the centre and surface of a single, undoped bulk is shown in Fig. 6(a), whereas the trapped field at the centre and surface of a two-sample-stack of undoped bulks is shown in Fig. 6(b)^40^. A trapped magnetic flux density of 2.47 T and 2.44 T was recorded at 5 K at the centre of the top and bottom surfaces of the 5% B_4_C (MPIG) bulk superconductor, respectively, confirming a high degree of uniformity in the sample microstructure [Fig. 6(a)]. This value of trapped field represents an increase of 16% over the one measured for the undoped MPIG sample, which decreases with temperature, reaching 0 T at 36.2 K. The two-sample stack of 10% B_4_C (MPIG) bulk superconductors trapped 3.75 T and 2.7 T at the centre and top surface at 5 K, respectively, which represents a 25% increase over the trapped field observed in an undoped MPIG bulk of same geometrical configuration [Fig. 6(b)], which decreased relatively rapidly to reach 0 T at 34.6 K. It can be concluded, therefore, that the rate of change of trapped field with temperature (*−dB/dT*) decreases with C-doping. The observed increase in trapped field from the undoped MPIG to 10% B_4_C (MPIG) correlates, clearly, with higher *J*_*c*_ at lower temperature and vice versa, and is due directly to increased C-doping in the bulk composition [Fig. 5(b)].

To date, much of the enhancement in trapped field in MgB_2_ has been achieved by grain refinement. For instance, Fuchs *et al*. and Sugino *et al*. used nano-scale Mg and B powders produced by high energy ball milling to synthesize MgB_2_ in an attempt to maximise grain boundary density^19,55^. These samples trapped maximum fields of 5.4 T (12 K) and 3.72 T (5 K) respectively. Similarly, Naito *et al*. observed that the addition of Ti (nominal composition Mg_1−*xi*_Ti_*xi*_B_2_) resulted in the formation of a thin layer of TiB_2_ on the MgB_2_ crystal that pinned the grain boundaries of MgB_2_ and inhibited grain growth^56^. Such a TiB_2_ layer was also thought to form a vortex point pinning site. On the contrary, the increase in trapped field of Mg(B_1−*xi*_C_*xi*_)_2_ bulk superconductors observed in the present study originates from atomic level intra-band scattering associated directly with C-doping. In addition, the increase has been achieved by the simple addition and mixing of B_4_C with B powder, and without the use of expensive techniques such as high energy ball milling. This demonstrates that C-doping via B_4_C in the IG process is an effective way of fabricating high performance MgB_2_ bulk superconductors that can potentially generate high magnetic fields.

Finally, a five-sample stack arrangement was constructed in order to investigate the effect of thickness on the trapped field, as shown in the inset to Fig. 6(c), which is significant given the potential of bulk MgB_2_ for application as a coaxial cylindrical superconducting magnet (which is consistent with this geometrical sample arrangement). A Hall sensor was placed on top of the 5-sample stack (H2) and another was sandwiched between the bulk superconductors (H1). A maximum trapped flux density of 4.15 T and 2.2 T was measured on H1 and H2 at 7.5 K, of which the former is the highest trapped field observed to date in MgB_2_ bulk samples fabricated under ambient pressure. Measurement (ii) was repeated with an external field of 6 T, which was observed to have very little effect on the magnetic flux density measured at both in the centre and surface of the two-sample stack (not shown here). The trapped field of 2.32 T (H2 extrapolated to 5 K) represents a ~10% increase in the magnetic field compared to that observed in a single bulk sample. Interestingly, 4.3 T (H1 extrapolated to 5 K) represents a ~15% increase with respect to trapped field at the centre of 10% B_4_C (MPIG) at 5 K. This suggests that such a stack arrangement could be used to generate high trapped fields in bulk MgB_2_. Simulation studies, such as that reported in ref.^57^, could be very useful to understand the distribution of magnetic flux within such a sample arrangement.

#### Flux Creep

**Figure 7:**
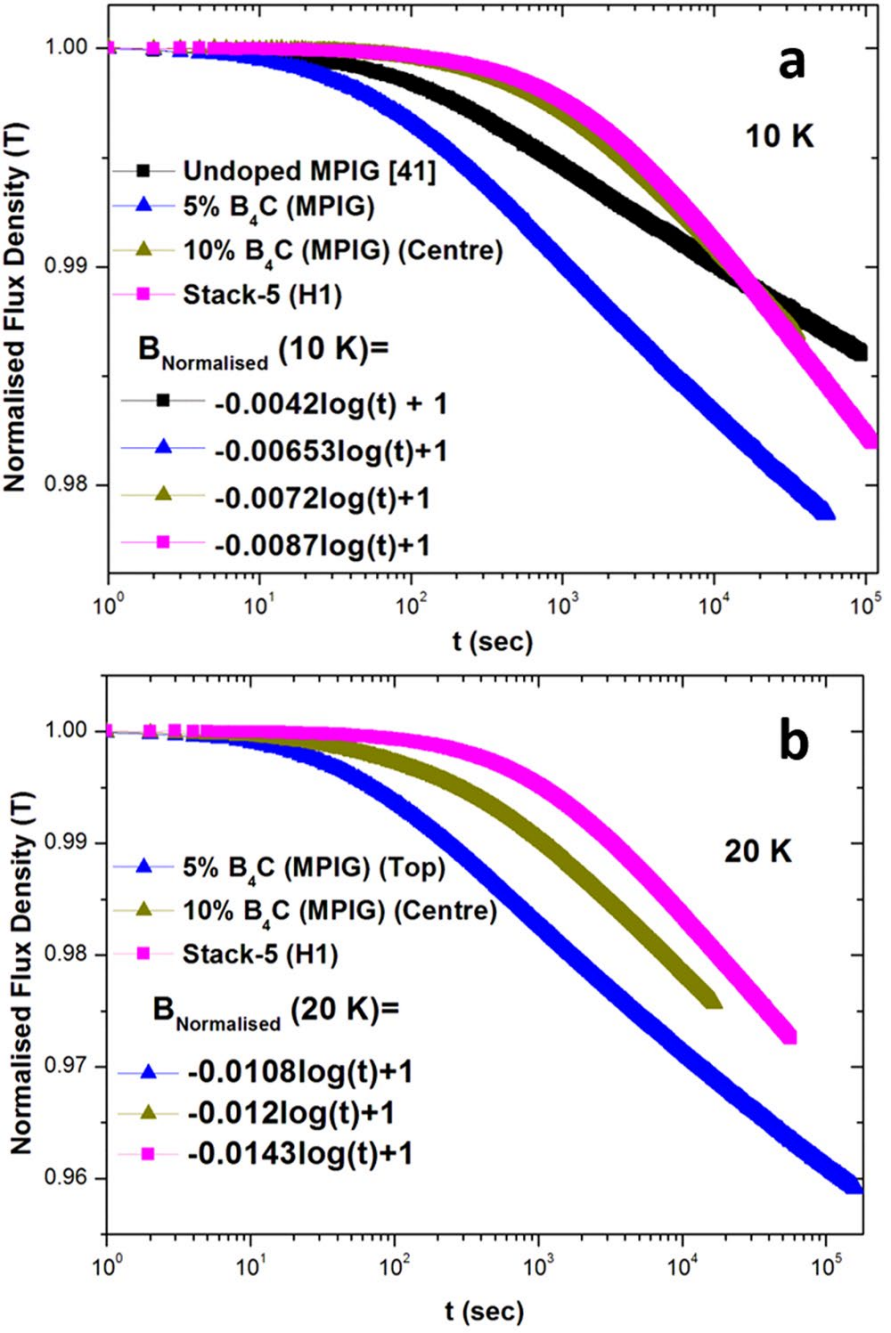
Time dependence of normalised field in undoped, 5% B_4_C (MPIG) (top of single bulk), 10% B_4_C (MPIG) (centre of stack), Stack-5 (H1) at (**a**) 10 K and (**b**) 20 K, respectively.

Loss of flux from the superconductor occurs when the Lorentz force exceeds the flux pinning force, in which case when *J* > *J*_*c*_. In addition, flux also ‘leaks’ at *J* < *J*_*c*_ due to thermally activated flux motion. MgB_2_, in particular, exhibits a low field decay rate compared to its high temperature superconductor counterparts at a given normalised temperature^58^. This is important from application perspective, since high temporal stability is desired in devices for Magnetic Resonance Imaging/Nuclear Magnetic Resonance, for example. Yamamoto *et al*. measured a low decay of 1.7%^59^ in peak trapped field after 3 days, while Naito *et al*. noted a small decay of 2% after 40 hrs of field removal^56^. The stability of peak trapped magnetic field at the centre in the MgB_2_ bulk samples was investigated by measuring the magnetic field relaxation at 10 K and 20 K after removal of the magnetizing field (Shown in Supplementary Figs 3 and 4). The time dependence of normalised trapped field at 10 K and 20 K for various samples is shown in Fig. 7(a,b), respectively. All the curves exhibit typical logarithmic decay (with the relevant equations given in the insets) with time (*t* > 100 s), although the variation in flux creep was observed to be non-logarithmic for *t* < 100 s. The decrease in normalised trapped field after one day at 20 K was observed to be significant greater than that observed at 10 K. For example, 5% B_4_C (MPIG) showed a 2.3% and 3.8% reduction in trapped flux at 10 K and 20 K, respectively after 1 day. This can be explained by following equation from the Anderson-Kim model^60^:

4 $${\rm{\nu }}={{\rm{\nu }}}_{0}{{\rm{e}}}^{\frac{-{{\rm{U}}}_{0}}{{{\rm{k}}}_{{\rm{B}}}{\rm{T}}}}$$

where *ν* is the jump attempt frequency from one pinning site to another, *U*_*o*_ is the activation energy (i.e. height of the potential well) for the de-pinning of flux line and *k*_*B*_ is Boltzmann’s constant. The trapped field is expected to decay with time since the flux jump frequency increases with temperature. Interestingly, the creep rate was also found to increase with C-doping, although stronger grain boundary pinning is expected for these samples.

## Conclusion

C-doped MgB_2_ samples were prepared by IG technique using B_4_C and SiC as sources of C. Samples fabricated using B_4_C showed a more homogeneous C distribution compared to those containing SiC. This is attributed to the higher reactivity of B_4_C with Mg and an atomically uniform distribution of C in the bulk microstructure. Various Mg(B_1-*xi*_C_*xi*_)_2_ phases in the B_4_C containing samples were identified using XRD and their lattice parameters were calculated. These samples showed significant enhancement in *J*_*c*_, particularly at lower temperature and higher fields. We account for this as being due to the generation of lattice strains and a loss of crystallinity in the MgB_2_ phase accompanied by the effects of C-doping. Analysis of the pinning force also suggested the possibility of a contribution to enhanced *J*_*c*_ from point pinning. A significant increase in trapped field was observed in C-doped MgB_2_ bulk superconductors. The trapped field obtained (4.15 T) in a stack of five stacked of bulk samples is the highest reported to date for MgB_2_ bulk superconductors synthesized under ambient pressure conditions. Finally, we note that the finer particle size of B_4_C is likely to yield more efficient and uniform C-doping, without the formation of MgB_2_C_2_ and leaving residual B_4_C. This, together with nano-sized boron powder, would potentially yield an optimum combination of enhanced grain boundary pinning and increased *H*_*c2*_, which could extend significantly the performance boundaries of bulk MgB_2_.

## Electronic supplementary material

Supplementary Information
