# Supplementary material for: High Trapped Fields in C-doped MgB2 Bulk Superconductors Fabricated by Infiltration and Growth Process
Source: Sci Rep. 2018 Sep 6;8:13320. doi: 10.1038/s41598-018-31416-3 (PMC6127330; doi:10.1038/s41598-018-31416-3)
Supplement: Supplementary file 1 — Supplementary Information [file 41598_2018_31416_MOESM1_ESM.docx]

**High Trapped Fields in C-doped MgB_2_ Bulk Superconductors Fabricated by Infiltration and Growth Process**

**A. G. Bhagurkar^a*^, A. Yamamoto^b^, L. Wang^c^, M. Xia^c^, A. R. Dennis^d^, J. H. Durrell^d^, T. A. Aljohani^e^, N. H. Babu^a^, D. A. Cardwell^d^**

^a^Brunel Centre for Advanced Solidification Technology, Brunel University London, Uxbridge,

UB8 3PH, UK

^b^Department of Applied Physics, Tokyo University of Agriculture and Technology, 2-24-16 Nakacho, Koganei, Tokyo 184-8588, Japan

^c^Shanghai Jiao Tong University, 800 Dong Chuan Road, Shanghai, 200240, China

^d^Department of Engineering, University of Cambridge,

Trumpington Street, CB2 1PZ, UK

^e^National Centre for Advanced Materials, King Abdulaziz City for Science and Technology,

Riyadh 11442, Saudi Arabia


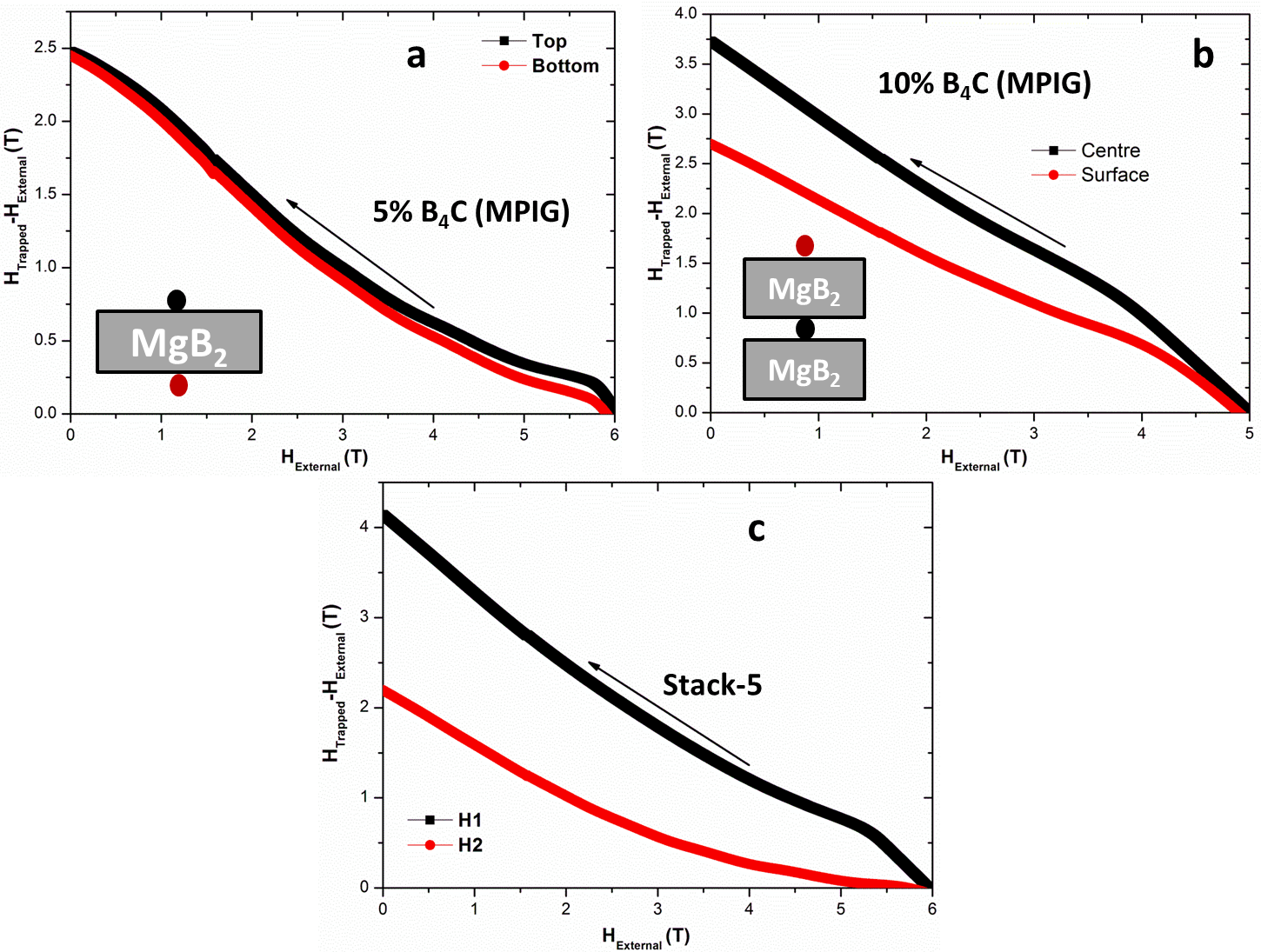
Figure 1. Difference between trapped magnetic flux density and external field as a function of external field at 5 K for figure (a) a single 5% B_4_C (MPIG), (b) two sample stack of 10% B_4_C (MPIG) and (c) Stack-5.

**
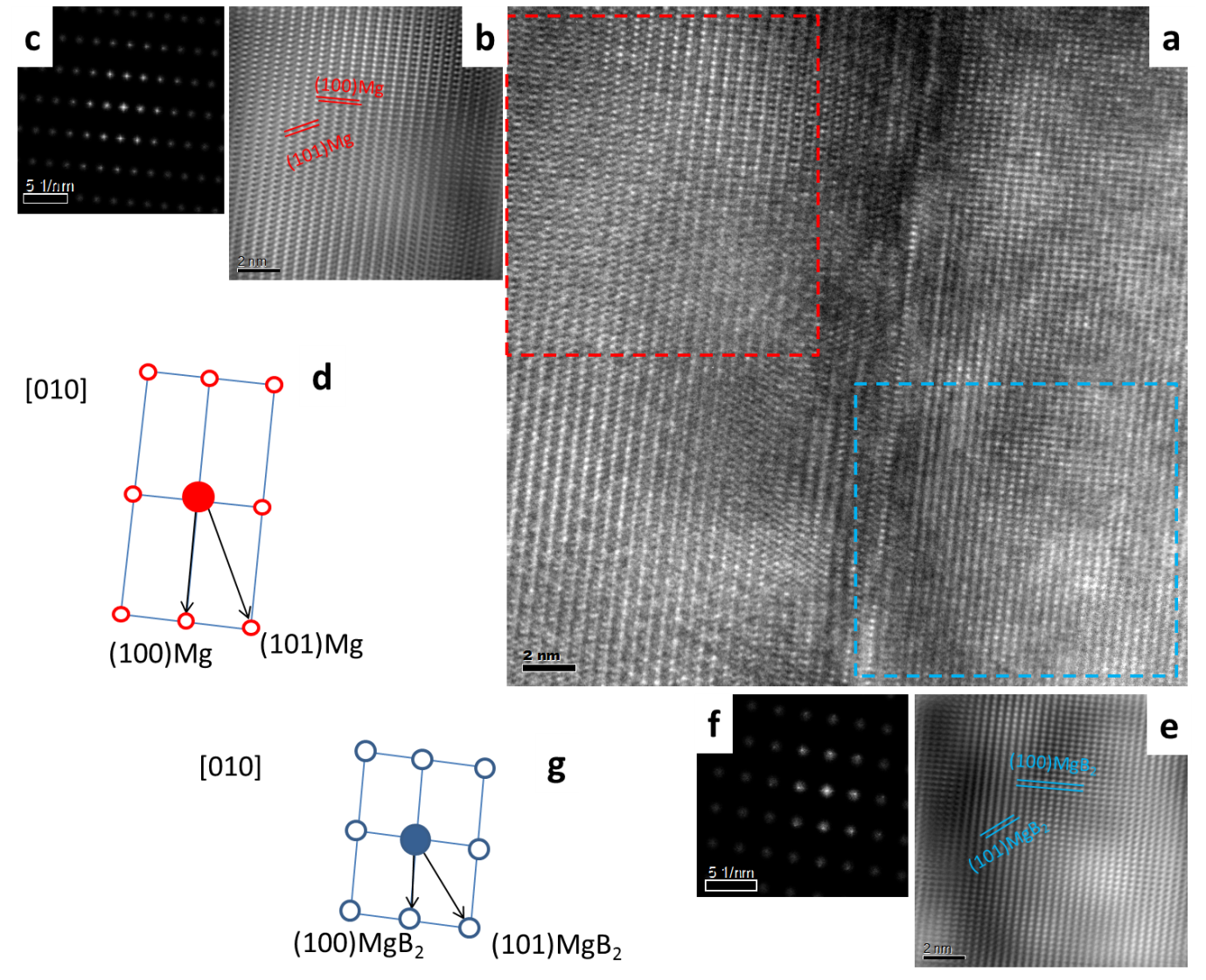
**

**Figure 2. (a) HRTEM image of C-doped sample (*x* = 10) with corresponding FFT patterns for (c) Mg (marked as red rectangle) and (f) for right side (marked as blue rectangle). The IFFT images reveal the lattice image of two crystals (b) Mg and (e) MgB_2_ phase. The schematic index for each phase are marked in (d) for Mg and (g) for MgB_2_.**


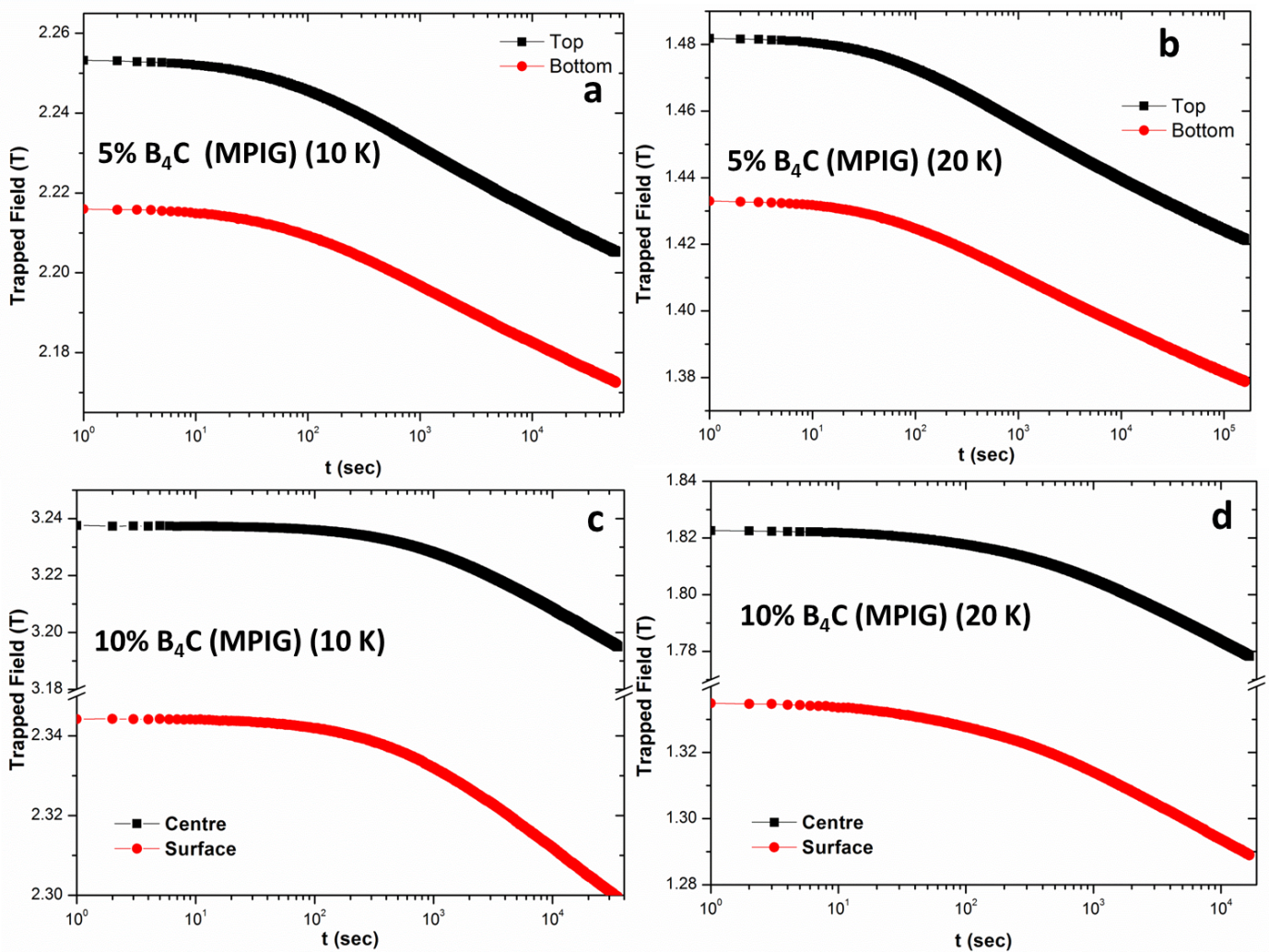


Figure 3. Time dependence of trapped magnetic flux density in 5% B_4_C (MPIG) on top and bottom at (a)10 K and (b)20 K. (c) and (d) shows field decay in centre and surface of two sample stack of 10% B_4_C (MPIG) bulks at 10 K and 20 K respectively.


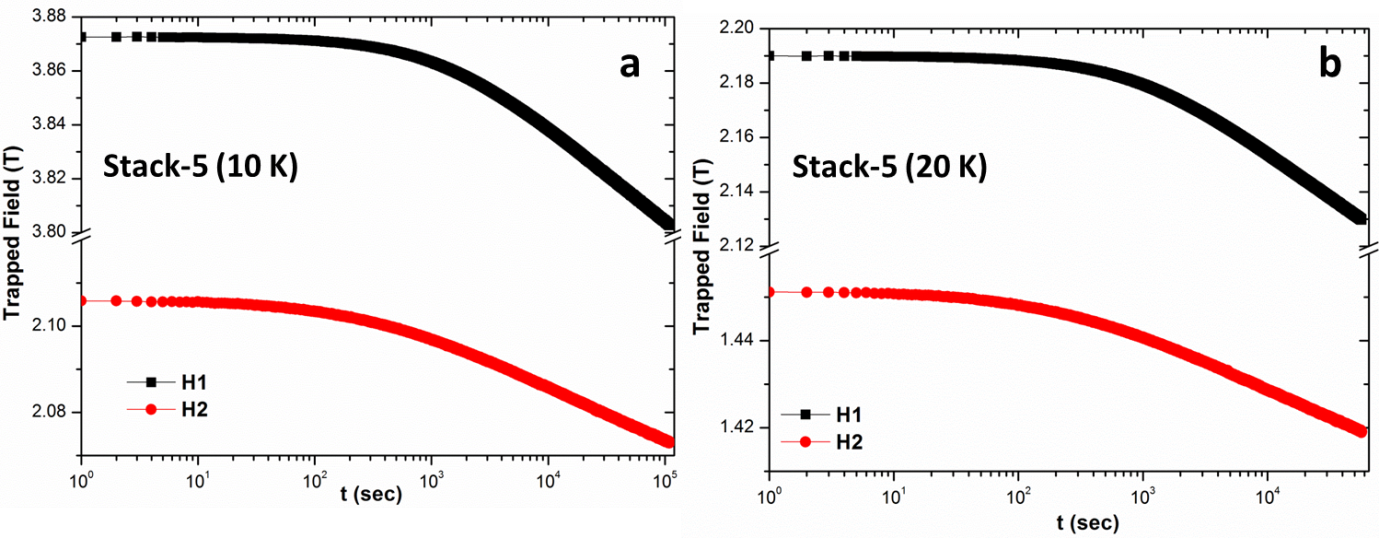
Figure 4. (a) and (b) showing the variation of trapped field in stack-5 obtained at hall sensors positioned at H1 and H2 at 10 K and 20 respectively.
